# Differential DNA Methylation in Purified Human Blood Cells: Implications for Cell Lineage and Studies on Disease Susceptibility

**DOI:** 10.1371/journal.pone.0041361

**Published:** 2012-07-25

**Authors:** Lovisa E. Reinius, Nathalie Acevedo, Maaike Joerink, Göran Pershagen, Sven-Erik Dahlén, Dario Greco, Cilla Söderhäll, Annika Scheynius, Juha Kere

**Affiliations:** 1 Department of Biosciences and Nutrition, Karolinska Institutet, Stockholm, Sweden; 2 Department of Medicine Solna, Translational Immunology Unit, Karolinska Institutet, Stockholm, Sweden; 3 Institute of Environmental Medicine, Karolinska Institutet, Stockholm, Sweden; 4 Science for Life Laboratory, Stockholm, Sweden; 5 Department of Medical Genetics, University of Helsinki and Folkhälsan Institute of Genetics, Helsinki, Finland; Cleveland Clinic Foundation, United States of America

## Abstract

Methylation of cytosines at CpG sites is a common epigenetic DNA modification that can be measured by a large number of methods, now even in a genome-wide manner for hundreds of thousands of sites. The application of DNA methylation analysis is becoming widely popular in complex disorders, for example, to understand part of the “missing heritability”. The DNA samples most readily available for methylation studies are derived from whole blood. However, blood consists of many functionally and developmentally distinct cell populations in varying proportions. We studied whether such variation might affect the interpretation of methylation studies based on whole blood DNA. We found in healthy male blood donors there is important variation in the methylation profiles of whole blood, mononuclear cells, granulocytes, and cells from seven selected purified lineages. CpG methylation between mononuclear cells and granulocytes differed for 22% of the 8252 probes covering the selected 343 genes implicated in immune-related disorders by genome-wide association studies, and at least one probe was differentially methylated for 85% of the genes, indicating that whole blood methylation results might be unintelligible. For individual genes, even if the overall methylation patterns might appear similar, a few CpG sites in the regulatory regions may have opposite methylation patterns (i.e., hypo/hyper) in the main blood cell types. We conclude that interpretation of whole blood methylation profiles should be performed with great caution and for any differences implicated in a disorder, the differences resulting from varying proportions of white blood cell types should be considered.

## Introduction

DNA methylation is the covalent addition of a methyl group in the position 5 of a cytosine (C) when this nucleotide occurs next to a guanine (G) forming a CpG site. There are around 28 million CpG sites in the human genome. Depending on the chromosomal region, cell type, developmental stage, alleles and parent-of-origin, a CpG site can be methylated, unmethylated or hemi-methylated. DNA methylation is involved in regulation of transcriptional repression and gene silencing. Together with other epigenetic mechanisms, DNA methylation functions as a switch that turns relevant genes on and off, a mechanism that is crucial in development, differentiation and homeostasis [1]. Certain CpG sites are highly methylated in hematopoietic progenitors but become unmethylated during differentiation [2], [3], [4]. There is also a small number of genes that gain cell specific methylation when the embryonic stem (ES) cells differentiate into the three germ layers [5]. The search for those methylated and/or unmethylated CpG sites that may categorize tissues and cell populations have been under extensive research for more than two decades [6], [7]. It is known that cell specific DNA methylation patterns convey “cell memory”, which is transmitted to the progeny by mitosis [8]. Therefore, every differentiated cell type has CpG sites that are specifically methylated or unmethylated for that specific lineage but not for others [2], [9].

There is currently extensive research ongoing aiming at the identification of specific changes in DNA methylation that may contribute to human diseases. Alterations in DNA methylation have been shown to cause monogenic disease such as Rett syndrome [10], and mediate genomic instability, silencing of tumor-suppressor genes and hyper-methylation of CpG island shores that may lead to the inception and progression of cancer [11]. Results of genome wide association studies together with the marked increase in the prevalence of several complex diseases during the last decades, for example asthma and allergy, suggests that other mechanisms such as epigenetics, including DNA methylation, may also be involved [12], [13]. These hypotheses have been supported by the differential effect of genetic polymorphisms depending on parent-of-origin [14], DNA methylation differences in disease-discordant monozygotic twins [15], [16], [17], differences in DNA methylation related to environmental exposures [18], [19], and DNA methylation differences in affected *versus* non-affected tissues [20], [21]. Given the limitations to obtain large number of samples from affected tissues, blood is an attractive, easy and available source of DNA. Studies suggest that DNA methylation differences can be detected in the blood of patients with cancer, even for solid tumors [22], [23], [24]. There is an increasing number of publications comparing differences in DNA methylation in whole blood between cases and controls for complex diseases [25], [26], [27], [28]. Topperoff *et al* found a specific methylation pattern in whole blood from patients with Type 2 diabetes that could be detected prior the onset of the disease [27]. In addition, differences in DNA methylation were identified in leukocytes of mothers having children with congenital heart defects [25]. Importantly, cell heterogeneity may act as a confounder when measuring DNA methylation in whole blood and the possibility to adjust for differential cell counts is being explored [27]. However, it is still unclear whether this strategy suffices to correct for inter-individual variation, lineage relationships (myeloid *versus* lymphoid), and potential effects of prominent methylation differences in less frequent cell populations (e.g. B cells, eosinophils, T regulatory cells).

The aims of this study were 1) to identify differentially methylated CpG sites globally in purified blood cells in connection with their genomic distribution; 2) to identify genes harboring the differentially methylated CpG sites and their connections with cell lineage and functions; and 3) to compare the DNA methylation profiles in a selection of candidate genes for complex inflammatory diseases among blood cell populations.

## Results

### General description of cell populations

**Figure 1:**
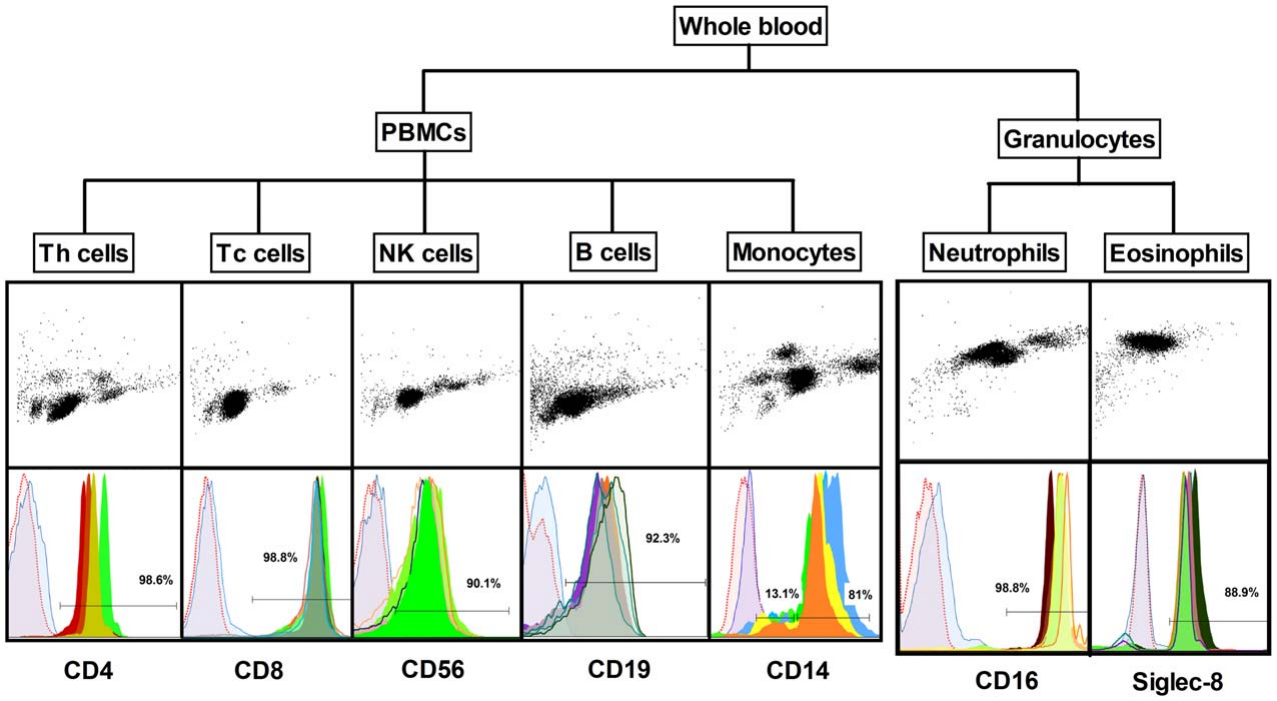
Schematic presentation of the isolation protocol and purity of the cell populations as measured by flow cytometry. Peripheral blood mononuclear cells (PBMC) and granulocytes were obtained by density gradient centrifugation and seven cell populations were purified by magnetic sorting. Upper panel shows forward and side scatter which confirmed cell morphology and granularity. The lower panel shows the overlay of cell surface markers for all the six donors. The purities of the cell populations were highly similar among all the six donors. Th cells  =  CD4^+^ T cells, Tc cells  =  CD8^+^ T cells, NK cells  =  CD56^+^ NK cells, B cells  =  CD19^+^ B cells, Monocytes  =  CD14^+^ monocytes. Data analyses are based on the comparison of all cell populations to whole blood.

Six healthy male blood donors, age 38±13.6 years, were included in the study. From each individual, global DNA methylation levels were analyzed in whole blood, peripheral blood mononuclear cells (PBMC) and granulocytes as well as for seven isolated cell populations (CD4^+^ T cells, CD8^+^ T cells, CD56^+^ NK cells, CD19^+^ B cells, CD14^+^ monocytes, neutrophils, and eosinophils, Figure 1). The differential cell count in whole blood was similar for all six donors as well as the purities of sorted cell populations as determined by flow cytometry (Figure S1, Table S1). The purities based on surface cell markers ranged from lowest 81.8±18.8% for eosinophils to highest 97.8±1.7% for neutrophils (Table S2).

### DNA methylation differences reflect cell lineages rather than inter-individual differences

**Figure 2:**
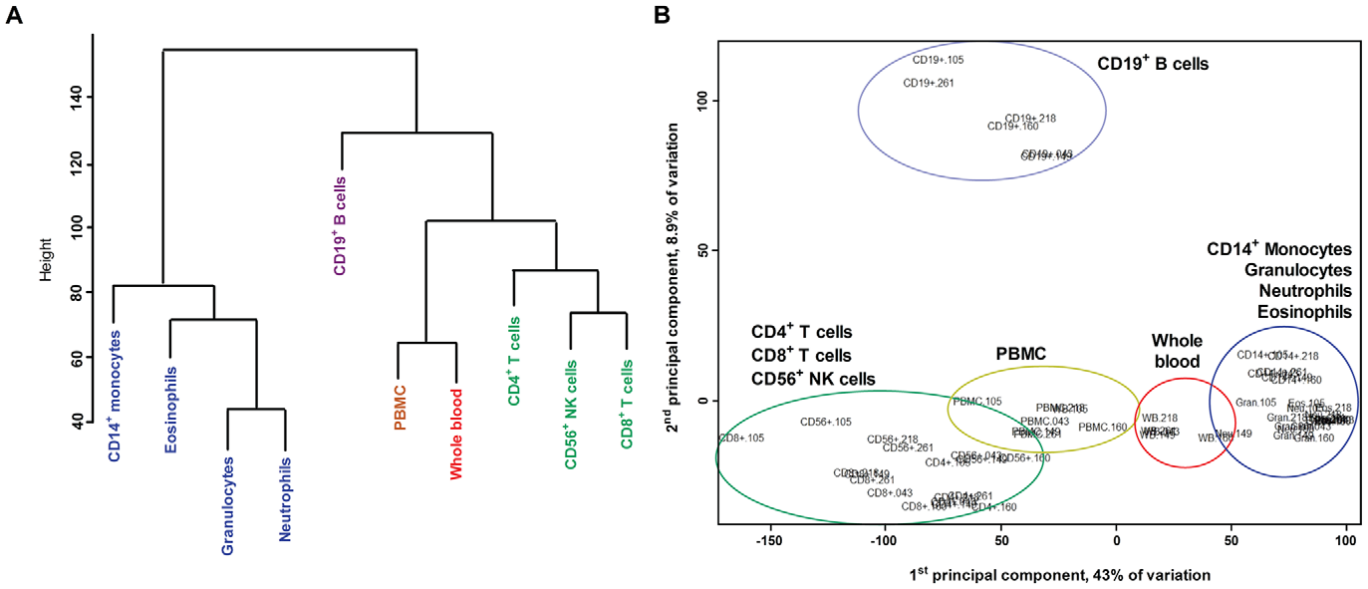
Clustering of cell populations in blood with regard to global DNA methylation. A) Hierarchical tree presenting the relationship between cell populations based on median M-values from six donors. B) Principal component analysis of each individual sample showing specific clustering based on cell population.

Global DNA methylation was analyzed using the Illumina Infinium 450K microarray platform [29]. When comparing all the cell populations against each other, there were large variations in the number of differentially methylated CpG sites (Table 1). These differences were reflected in a clear clustering of all studied cell populations according to their expected hematopoietic lineage when using the median M-value from each cell population (Figure 2A). Furthermore, a principal component analysis including all individual observations (n = 60) showed that the DNA methylation patterns differ more between cell populations than between individuals (Figure 2B). The distribution of the mean M-values and the standard deviations were similar for all cell populations, further supporting the low inter-individual variance in this study (Figure S2). Surprisingly, CD19^+^ B cells clustered separately from the other lymphoid-derived cells (Figure 2A and B).

**Table 1:** Differentially methylated probes based on M-values in each studied cell population in blood compared to each other.

	PBMC	CD4^+^ T cells	CD8^+^ T cells	CD56^+^ NK cells	CD19^+^ B cells	CD14^+^ Monocytes	Granulocytes	Neutrophils	Eosinophils
**Whole Blood**	23226	58980	125713	92238	97377	41063	27365	28581	52431
**PBMC**		23168	76847	33642	58485	98771	94601	87348	104631
**CD4^+^ T cells**			45015	38396	73318	115235	109933	106881	120620
**CD8^+^ T cells**				20629	98306	161524	164184	182881	192075
**CD56^+^ NK cells**					78880	157180	154816	146413	158727
**CD19^+^ B cells**						143376	147665	134890	150548
**CD14^+^** **monocytes**							16011	17440	30949
**Granulocytes**								169	22423
**Neutrophils**									16241
**Eosinophils**									

PBMC – Peripheral blood mononuclear cells. Differentially methylated probes were defined by a linear model using the M-values.

**Figure 3:**
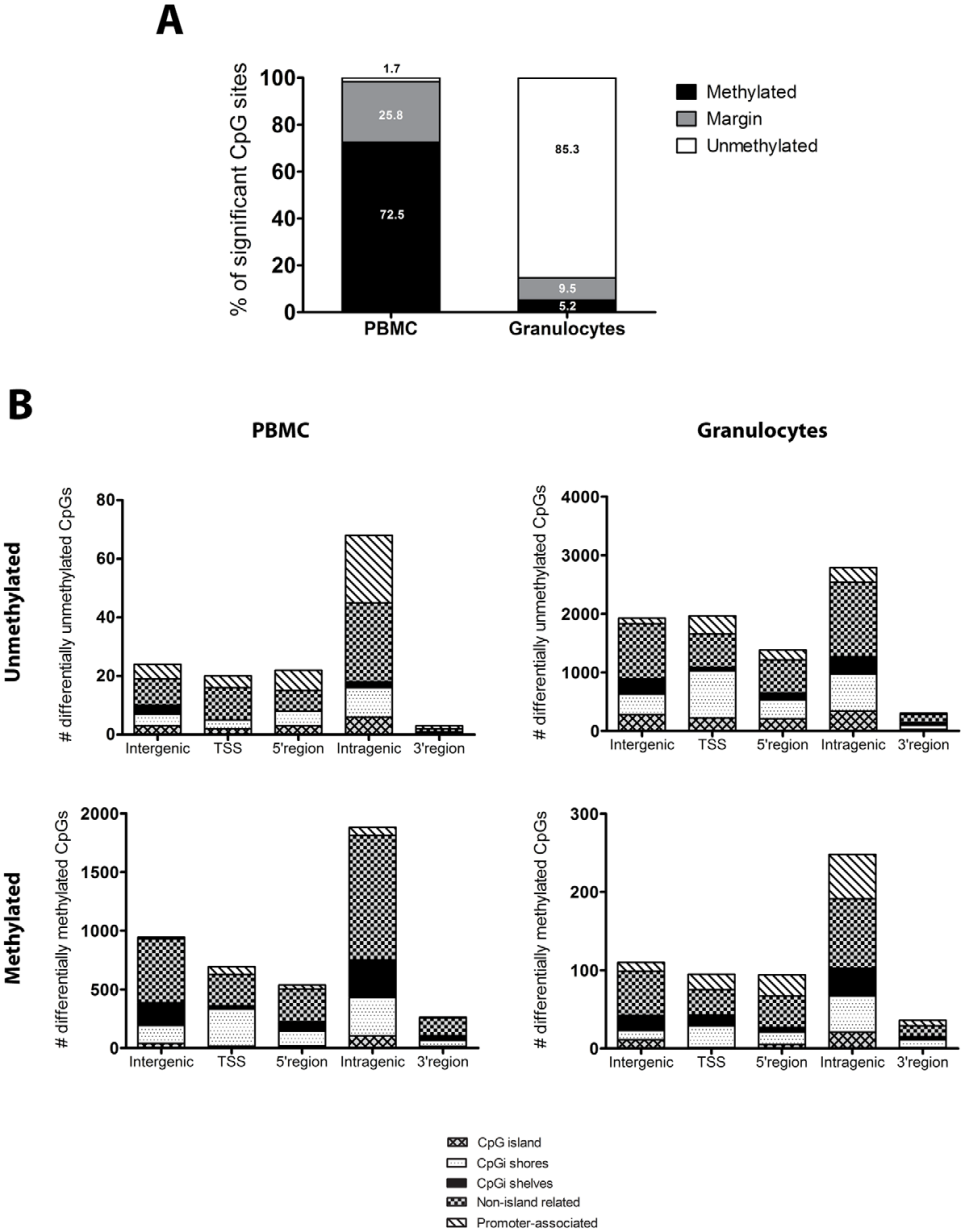
Differentially methylated CpG sites compared between peripheral blood mononuclear cells (PBMC) and granulocytes. A) The distribution of probes in the two populations defined by the calls unmethylated, margin and methylated. Data is based on a linear model comparing the two principal cell populations to whole blood using M-values. The data was then subjected to a gamma fit model in order to group the data into the defined calls. The value within the bars represents the percentage of the distribution within each population. B) The genomic distribution of probes showing significantly differential methylation compared to whole blood for PBMC and granulocytes. Genomic regions were defined according to the UCSC RefGene group (included in the Illumina annotation data). Probes are divided on the unmethylated and methylated state according to the gamma fit model for both PBMC and granulocytes. Intergenic  =  site not annotated in a gene, TSS  =  transcription start site at 200–1500 bp, 5′ region  = 5′UTR and 1st exon, Intragenic  =  gene body including introns and exons and, 3′ region  = 3′UTR. UTR – untranslated region.

Since we were interested in whether whole blood is a valid source for DNA methylation analyses, all the following analyses were restricted to compare each cell population to whole blood (see methodology section). In order to select the CpG sites of highest interest, we also restricted our focus on the significantly differentially methylated sites with a differential methylation call, as defined by the gamma fit model (“unmethylated”, “marginal”, “methylated”). This analysis revealed that in the myeloid cell populations, most of the CpG sites were unmethylated while for the lymphoid populations, the majority was either marginal or methylated (Table 2 and Figure 3A). When comparing the two major leukocyte fractions in whole blood, PBMCs and granulocytes, there was a large overlap of differentially methylated CpG sites based on the median M-value (Figure S3). However, when looking at the differentially methylated sites showing either unmethylated or methylated state separately, the overlaps were reduced (39% overlap for all, 4% for unmethylated and 12% for methylated CpG sites, Figure S3). Furthermore, the genomic distribution of the differentially methylated sites was studied in each cell population. This revealed that most of the statistically significant unmethylated and methylated CpG sites were located in intragenic regions (gene bodies) and not associated with CpG islands. There were no differences in this distribution between PBMCs and granulocytes (Figure 3B) or for any of the purified cell types (data not shown). Also, the data confirmed that CpG island shores are more commonly harboring differentially methylated CpG sites compared to CpG islands and CpG island shelves.

**Table 2:** Differentially methylated probes for each cell population in comparison to whole blood.

	M-value comparison	Comparison on calls*	Absolute variation in comparison to whole blood (M-values)**	Unmethylated	Marginal	Methylated
PBMC	23226	5693	1.2–11.1	1.7%	25.8%	72.5%
CD4^+^ T-cells	58980	18537	1.2–51.9	9.4%	34.5%	56.1%
CD8^+^ T-cells	125713	32948	1.2–93.2	6.8%	59.7%	33.4%
CD56^+^ NK cells	92238	23484	1.1–54.0	6.5%	58.4%	35.1%
CD19^+^ B cells	97377	23245	1.2–221.8	15.0%	55.7%	29.3%
CD14^+^ Monocytes	41063	12625	1.2–70.4	71.2%	13.2%	15.6%
Granulocytes	27365	8815	1.2–13.1	85.3%	9.5%	5.2%
Neutrophils	28581	10808	1.2–14.5	84.2%	10.1%	5.7%
Eosinophils	52431	17723	1.2–117.2	65.8%	23.3%	10.8%

PBMC-Peripheral blood mononuclear cells. Differentially methylated probes were defined by a linear model using the M-values. M-value is the log2 ratio of the intensities of methylated probe versus unmethylated probe, a measurement of how much more a probe is methylated compared to unmethylated [46]. *To extract the probes with largest difference in methylation, a gamma fit model was applied to M-values in order to define the three calls: “unmethylated”, “marginal” and “methylated”. Significant probes sharing the same call in the two compared populations were removed. **Variation is based on the estimate of the log2-fold-change corresponding to the effect obtained from the linear model, absolute M-values are presented. The percentages are based on the call distribution.

### Differential DNA methylation is related to genes involved in cell type-specific immune functions

**Figure 4:**
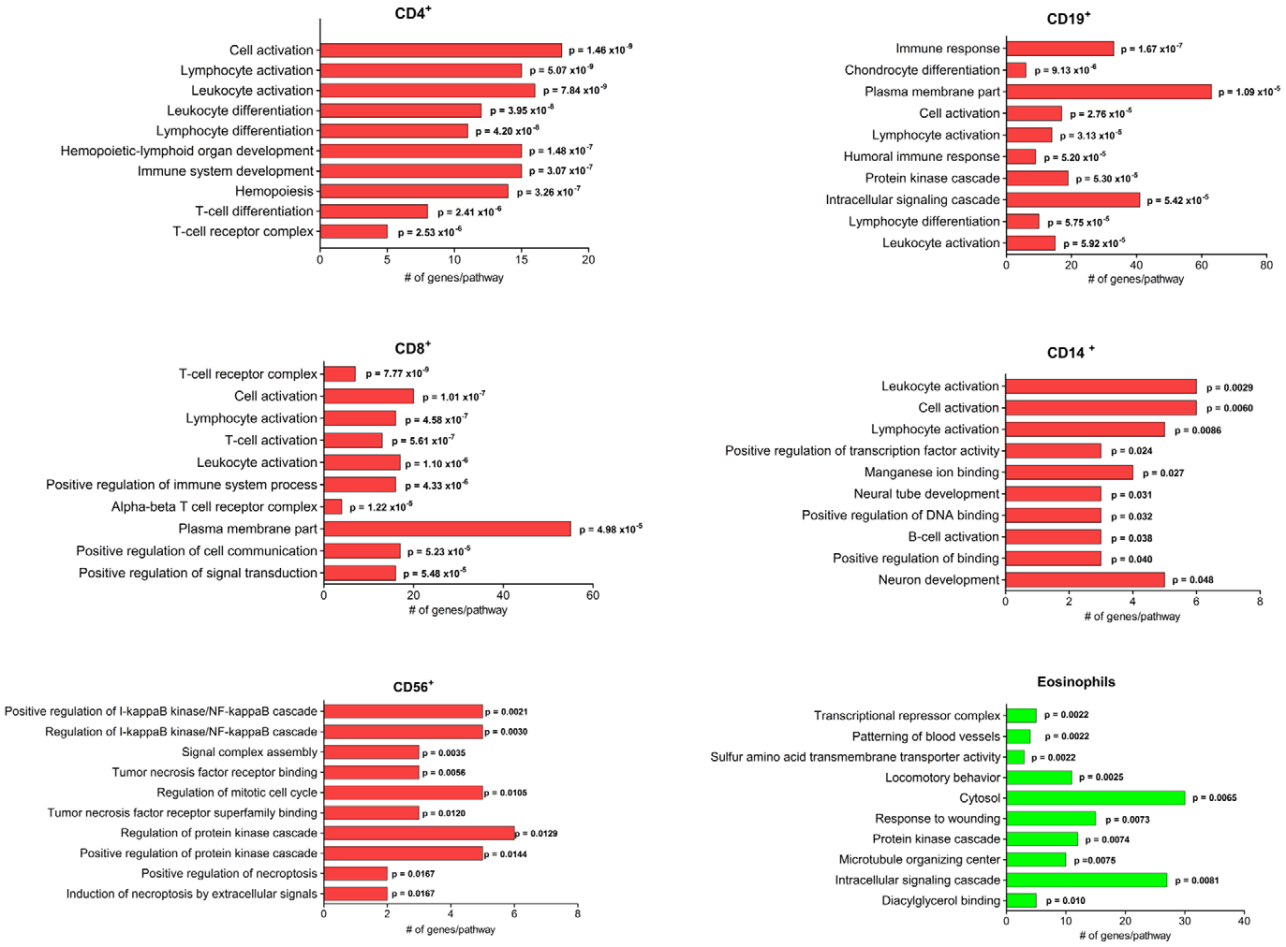
Gene ontology enrichment for isolated cell populations. Gene ontology was performed using DAVID (http://david.abcc.ncifcrf.gov) [47]. The human genome was used as background and the level of significance was set to p<0.05. The top ten enriched pathways are described for genes showing significantly differentially methylated probes in comparison to whole blood where the cell population shows unmethylated state and whole blood shows methylated state according to the gamma fit model. Red color indicates peripheral blood mononuclear cells (PBMC) and green color indicates granulocytes.

Functional enrichment analyses were performed for the genes showing significant unmethylated state in a specific cell population and methylated state in whole blood. Gene ontology enrichment suggested many expected cell specific categories for the cell types analyzed (Figure 4). CD4^+^ and CD8^+^ T cells showed the most cell specific enriched pathways including lymphocyte activation, T cell receptor complex category, leukocyte activation and cell activation. CD56^+^ NK cells had enrichment in pathways belonging to molecular signaling cascades. CD19^+^ B cells showed enrichment in the cell specific pathway humoral immune response and the category for plasma membrane part which included genes specific for antigen presentation. Also for CD14^+^ monocytes, pathways specific for the cells function such as leukocyte activation, lymphocyte activation and B-cell activation were enriched. For eosinophils, the enrichment was not directly related to cell type specific functions but rather more characteristic for general cell functions. With the criteria chosen for this analysis, *i.e.* comparing the cell specific methylation to that in whole blood, no probes were called unmethylated in CD16^+^ neutrophils and methylated in whole blood.

**Figure 5:**
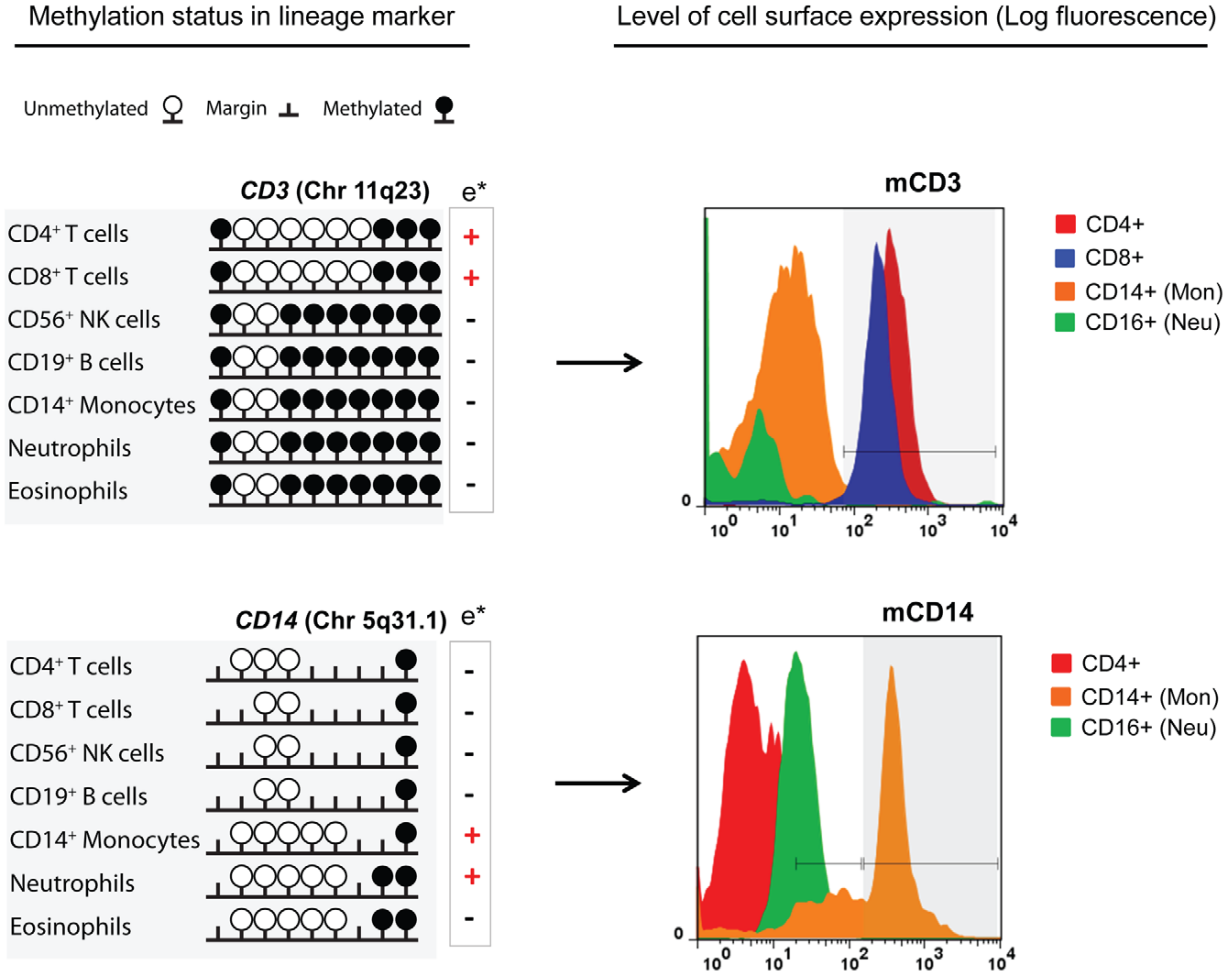
Differentially methylated sites are harbored by cell-specific genes but the connection between methylation status and surface expression depend on the gene and lineage. The surface expression of CD3 and CD14 according to the methylation status of the coding gene is presented. Demethylation of CpG sites was observed in T cells expressing the CD3 marker (upper panel). For CD14 the difference between positive and negative cells involved cell-restricted marginal status in a context of demethylation (lower panel). Histograms (right) represent the log fluorescence of the marker (*x*-axis) and the cell counts (*y*-axis). Peaks within the gray shaded gates represent positive populations. For CD14 two gates are presented, the “*low*” which include monocytes and a fraction of neutrophils and the “*high*” population of monocytes. m  =  membrane; e*  =  positive or negative cell expression of the marker according to the Human Leucocyte Differentiation Antigens (HLDA) Workshop and CD nomenclature (http://www.hcdm.org/Home/tabid/36/Default.aspx).

The functional role of the differentially methylated genes was further suggested by the cell-restricted patterns of DNA methylation in 30 genes according to their cell surface expression (based on the CD nomenclature). In general, demethylation of CpG sites was commonly found in lymphoid cells that were regarded as CD positive, while for myeloid cells the patterns were more complex and involved differences in single CpG sites or differences of marginal status. An example is presented in Figure 5, showing the methylation of the *CD3* and *CD14* genes in all the purified cells in connection with their membrane expression as determined by flow cytometry.

### Blood cell specific patterns of candidate genes in inflammatory complex diseases

**Figure 6:**
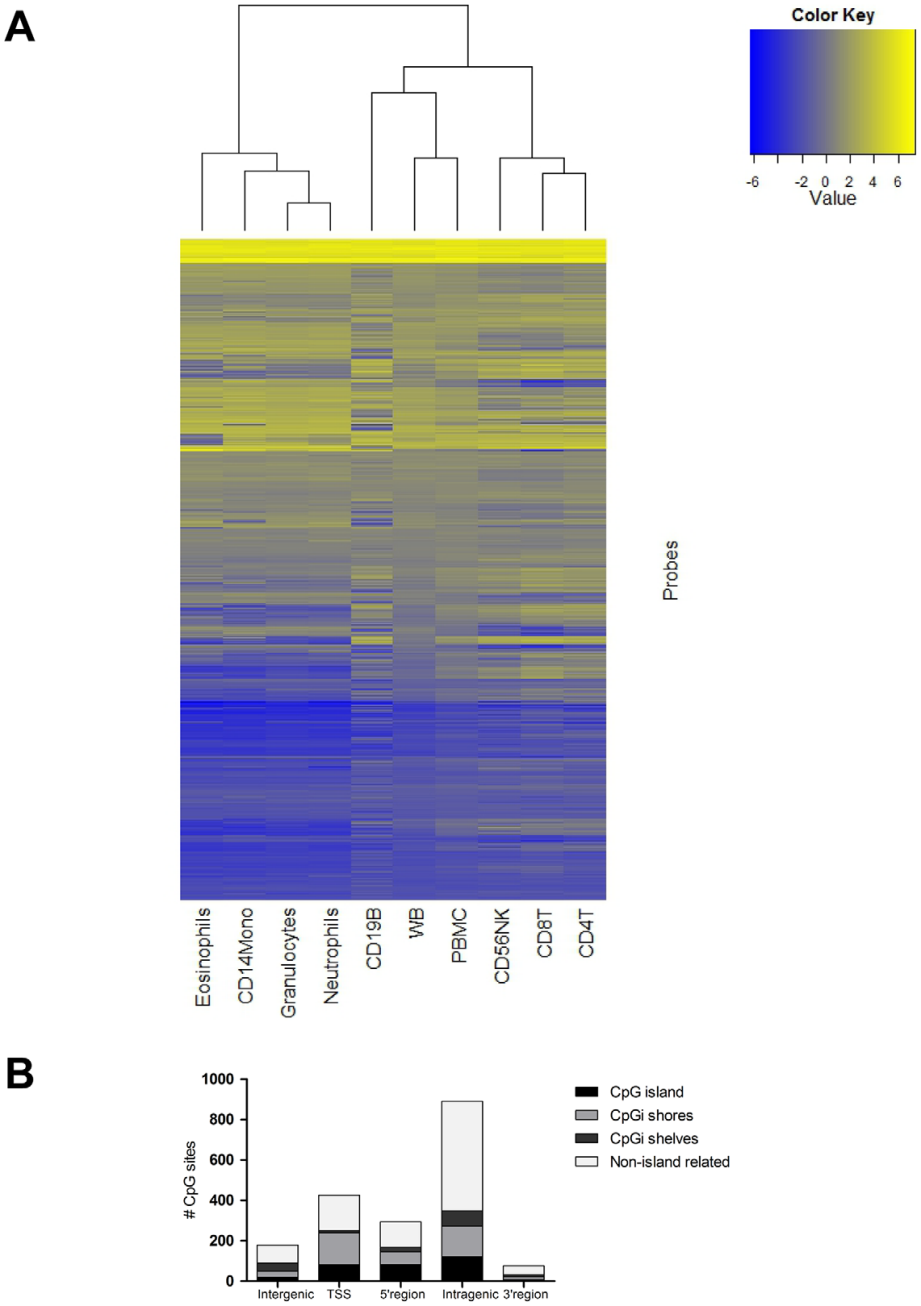
Differentially methylated CpG sites in candidate genes related to inflammatory diseases. A) Heatmap of 1865 probes representing 293 candidate genes for selected inflammatory diseases showing differential methylation in blood cell populations. Candidate genes for the diseases asthma, atopy, atopic dermatitis, inflammatory bowel disease, rheumatoid arthritis, systemic lupus erythematosus, Type 1 and Type 2 diabetes were selected from the Genome wide association study atlas (http://www.genome.gov/gwastudies/) [30]. The heatmap is based on median M-values. The M-value is calculated as the log2 ratio of the intensities of methylated probe versus unmethylated probe [46]. Blue color indicates low DNA methylation while yellow represents high DNA methylation. WB  =  whole blood, CD4T  =  CD4^+^ T cells, CD8T  =  CD8^+^ T cells, CD56NK  =  CD56^+^ NK cells, CD19B  =  CD19^+^ B cells, CD14Mono  =  CD14^+^ monocytes. B) The genomic distribution of the differentially methylated probes associated with inflammatory complex diseases according to the UCSC RefGene group (included in the Illumina annotation data). Intergenic  =  site not annotated in a gene, TSS  =  transcription start site at 200–1500 bp, 5′ region  = 5′UTR and 1st exon, Intragenic  =  gene body including introns and exons and, 3′ region  = 3′UTR. UTR – untranslated region.

Recent advances within the genetic field have produced a large number of candidate genes suggested to be involved in several complex diseases. In order to analyze to which extent differences in cell lineage may influence the DNA methylation profiles of known susceptibility genes for inflammatory diseases, a group of candidates were selected from the catalog of published genome-wide association studies (http://www.genome.gov/gwastudies/) [30]. The included diseases were asthma, atopy, atopic dermatitis, inflammatory bowel disease, rheumatoid arthritis, systemic lupus erythematosus, Type 1 and Type 2 diabetes. In total, 343 genes were selected and they were covered by 8252 probes. Of these probes, 1865 (22.6%, Table S3) were differentially methylated among cell types and they corresponded to 293 genes (85%, Figure 6A). CD19^+^ B cells showed the largest variation for these differentially methylated CpG sites. There was also a clear difference in the methylation patterns when comparing lymphocytes (CD4^+^ T cells, CD8^+^ T cells and CD56^+^ NK cells) to the myeloid cells (eosinophils, neutrophils and CD14^+^ monocytes). Again, most of the significantly differentially methylated CpG sites were located in intragenic regions (gene bodies, Figure 6B).

**Figure 7:**
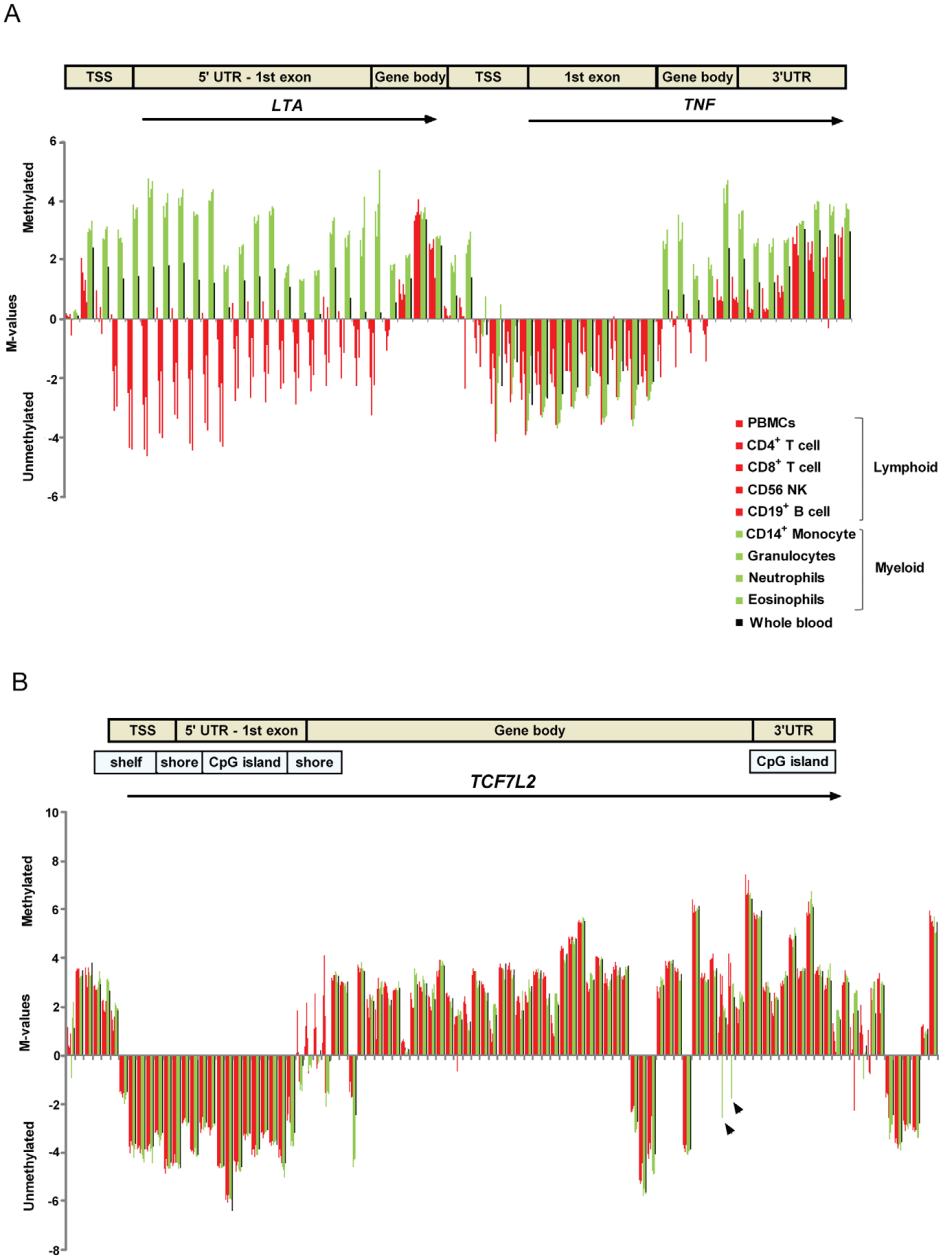
DNA methylation levels across the gene regions in purified cell populations for candidate genes. DNA methylation is defined by the M-values for each cell population at a given CpG site. The M-value is calculated as the log2 ratio of the intensities of methylated probe versus unmethylated probe and describes a measurement of how much more a probe is methylated compared to unmethylated [46]. Negative numbers represent unmethylated and positive numbers represent methylated. Every cell population correspond to a vertical bar which is listed from left to right as peripheral blood mononuclear cells (PBMC), CD4^+^ T cells, CD8^+^ T cells, CD56^+^ NK cells, CD19^+^ B cells, CD14^+^ monocytes, granulocytes, neutrophils, eosinophils and whole blood. Lymphoid cells are colored in red bars, myeloid cells are colored in green bars and whole blood is represented by black bars. A) the asthma candidate genes lymphotoxin alpha (*LTA*) and tumor necrosis factor (*TNF*), and B) the Type 2 diabetes candidate gene transcription factor 7-like 2 (*TCF7L2*), black arrows indicate regions with cell type specific pattern for monocytes. TSS  =  transcription start site at 200–1500 bp; UTR  =  untranslated region, gene body including introns and exons.

In depth analyses of the asthma associated genes encoding for lymphotoxin alpha (*LTA*) and tumor necrosis factor (*TNF*) on chromosome 6p21.3, showed that differentially methylated CpG sites are enriched in the promoter region (5′UTR, 1st exon) and differ according to lymphoid *versus* myeloid lineages (Figure 7A). Analyzing DNA methylation levels in whole blood for this region would lead to misleading conclusions since myeloid cells are methylated while lymphocytes are unmethylated. For the Type 2 diabetes candidate gene transcription factor 7-like 2 (*TCF7L2*) on chromosome 10q25.3, the methylation pattern is similar along the gene body but there are certain CpG sites that show unmethylated state in CD14^+^ monocytes only (see arrows, Figure 7B). These sites coincide with intron-exon boundaries in the 3′end of the gene. Methylation in the promoter CpG islands tends to be low and very similar among all the cell types and for those CpG sites, measurements in whole blood would reflect the methylation status across cell populations. Largest variations in DNA methylation can be seen along the CpG island shores and gene bodies (Figure 7A and B).

## Discussion

### Summary of main findings

Here, we present for the first time a genome wide methylation study covering approximately 450 000 CpG sites analyzed in selected cell populations in blood obtained from the same individual. Surprisingly, even though including only six healthy adult male donors with an age range between 25 and 60 years, we could see a clear clustering of the cell types according to their lineage rather than by individual. We also found large differences in DNA methylation between lymphoid and myeloid cells and identified many CpG sites that were differentially methylated between the purified cell populations. Interestingly, B cells showed the most unique profile, suggesting that studies in B cells or cell lines poorly reflect the overall blood cell methylation status. Many genes covered by these differentially methylated CpG sites were important for specific cell functions as highlighted by the gene ontology analysis and the cell-specific demethylation patterns according to their putative expression of surface markers. In addition, we found differential methylation in selected candidate genes for inflammatory diseases according to cell type. Importantly, regional differences occur within genes and may affect a cell fraction or just a single cell population. Our results indicate that caution should be exercised in interpreting results for a large fraction of genes, specifically immune-related, when analyzing whole blood DNA methylation results because the results will in many cases depend on the blood differential count.

### Myeloid *versus* Lymphoid lineage

Information on genome wide methylation patterns in human blood cell populations is scarce [31], [32], [33], [34]. Global analysis of CpG sites associated with promoter regions has revealed some cell specific differentially methylated sites by comparing monocytes and granulocytes [2] as well as for pooled purified blood populations [35]. In this study, we found enrichment of unmethylated regions in granulocytes while PBMCs were in general more methylated (Figure 3), confirming previous findings in bone marrow progenitors of these cells [9]. In the more common leukocytes, the neutrophils, approximately 85% of the differentially methylated sites were unmethylated. These cells do not divide and have an average lifespan of 5 days. The rapid turnover that is required to replace these cells may be due to the low requirements of the DNA methylation machinery, as suggested by hypomorphs for *Dnmt1* which have normal myelopoiesis but drastically reduced lymphopoiesis [36]. In monocytes, differentially methylated CpG sites were more frequently unmethylated (71%) and this may be related with the common granulocyte/macrophage progenitor that is shared with the granulocytes [4]. Comparative studies between CD34^+^ cells and differentiated monocytes suggested that DNA methylation could be a mechanism that silences the myeloid differentiation program [2]. In contrast, lymphocytes live from 10 days to several years, undergo positive and negative selection across different tissues and differentiate to subsets through cell divisions. It is possible that a state skewed towards more methylation in the lymphocytes may be favorable to keep cell memory during clonal expansion and to regulate the fate in different subsets of lymphocytes (e.g. effector *versus* memory cells).

### The B cell

The main role of the B cell is antigen presentation and production of antibodies, key components in the humoral immune response. Interestingly, we found that CD19^+^ B cells cluster separately from other lymphocytes and they show the largest number of differentially methylated CpG sites (Figure 2B and 5A). DNA methylation has previously been found to be of relevance in the allelic exclusion of the Fab light chains and in the V(D)J rearrangement of the immunoglobulin heavy chain locus [4]. However, we found many differentially methylated genes belonging also to other pathways indicating a global pattern separating B cells from other cell types in blood. Gene ontology categories suggested that in addition to the predictable humoral response genes, a large proportion of the enriched genes harboring differentially methylated sites are involved in internalization and presentation of antigens, which reflects a capital difference between B cells and T lymphocytes. Therefore, at the DNA methylation level, the B cells resemble an intermediate state between the monocyte and the T lymphocytes in part for their role as phagocytes and antigen presenting cells. Besides, the different developmental stages of circulating B cells such as transitional B cells, memory B cells and plasma cells, which have no counterparts in T cells, may have contributed to the particular B cell pattern.

### Regional effects of differential DNA methylation

Studies have shown that tissue-specific differentially methylated regions are highly conserved in mammals [37], [38] and located in crucial regulatory areas such as 5′UTRs and promoters as well as in gene bodies [39]. We showed that differentially methylated sites were more frequently located in gene bodies irrespective of whether or not they were methylated or unmethylated (Figure 3C and D) which is in agreement with Maunakea *et al*. They showed that a great proportion of tissue and cell type specific DNA methylation across gene bodies coincide with highly conserved sequences. Methylation within these gene bodies can then regulate intragenic promoter activity and therefore the alternative expression of splice variants [40]. Furthermore, recent findings in murine immune cells show that differential DNA methylation occurs primarily in CpG islands located inside gene bodies which are infrequently annotated with promoter CpG islands or CpG island shores [1]. The over-representation of differentially methylated sites in gene bodies may explain why strong CpG islands can be unmethylated and the gene remains transcriptionally inactive while promoters with low CpG content are more frequently methylated without precluding transcription [41]. The function of these sites within gene bodies as well as those located in non-coding RNAs remains to be investigated. Moreover, previous findings using whole genome methylation sequencing of PBMC [33] and B cells [32] support our finding that CpG sites in regions of high density such as CpG island and 5′UTR were more often unmethylated, while CpG sites located in introns, 3′ UTRs and repetitive elements were methylated.

### Blood cell specific patterns of candidate genes in inflammatory complex diseases

DNA methylation has gained a lot of interest within the studies of complex diseases. Some of the candidate genes obtained from genome wide association studies are currently being analyzed for DNA methylation in case-control datasets, but it is unknown whether they are amenable to be analyzed in whole blood. We analyzed a selection of candidate genes and found that 22% of the CpG sites within these genes were differentially methylated between cell types and B cells showed the largest variation (Figure 6A). Detailed examples of gene specific DNA methylation patterns in *LTA*, *TNF* and *TCF7L2* (Figure 7) showed that many regions within a gene may be unaffected by cell composition in whole blood and thus independent of cell counts. However, there are site specific changes that are highly influenced by cell type and these may be the sites of greatest interest to study in relation to disease. For example, the Type 2 diabetes candidate gene *TCF7L2* has a similar methylation pattern along the gene body but there are CpG sites showing unmethylated state in monocytes only (Figure 7B, see arrows). A study on tissue specific expression of *TCF7L2* isoforms showed that in blood, the isoform ‘ex7–8’ was only expressed in monocytes [42]. This might suggest that the regions we highlight could be regulating this tissue specific expression. The current lack of information regarding cell specificity imposes a limitation for the selection of CpG sites in comparative studies of DNA methylation using candidate approaches.

### Conclusions

The number of CpG sites that are differentially methylated between purified cells and whole blood and mixed fractions (PBMCs and/or granulocytes) generally reflects the frequency of the cell populations in both compartments, and consequently, adjustment of DNA methylation levels by differential cell counts may have some use in epidemiological studies. This is exemplified in the comparison between monocytes *versus* whole blood; or neutrophils *versus* granulocytes (Table 1) where both populations are frequent and prominently represented in each other. However, it should be considered that the relative contribution of the less abundant cell populations to the estimates in whole blood are not always in direct correlation with their frequency. For example the eosinophils are rare cells in the circulation and therefore the number of differentially methylated CpG sites compared to whole blood is masked by the partial overlap with the granulocyte compartment. Likewise, in many inflammatory diseases, the blood cells only weakly relate to the tissue specific pattern of inflammation. Together with the difficulties to draw *a priori* inferences on the cell-specific methylation for any given CpG site, these biological facts make candidate gene studies for DNA methylation in whole blood challenging.

In conclusion, this study provides important insights to the complex DNA methylation patterns defining specific cell types in peripheral blood. The clear differences between DNA methylation patterns in myeloid cells and lymphocytes as well as the distinct methylation profile of B cells highlight the difficulties of interpreting this type of analyses in whole blood. If performing DNA methylation analyses using whole blood, the cell specific pattern for the gene region of interest should therefore be evaluated beforehand.

## Materials and Methods

### Ethics Statement

This study was performed in accordance with the principles expressed in the Declaration of Helsinki. The protocol was approved by the local ethics committee at Karolinska Institutet. All individuals signed a written informed consent to participate in the study.

### Study participants

Purified blood cell populations were obtained from six male healthy blood donors (mean age 38±13.6 years), previously recruited within the MALF study [43]. The sampling was done between July and October 2010 at the Fridhemsplan Blood Bank, Stockholm. 450 mL of blood were collected from the median cubital vein into transfusion bags containing citrate phosphate dextrose (Terumo Corporation, Japan), stored at room temperature and processed within 24 hours.

### Purification of blood cell populations

Two aliquots of whole blood were initially taken from the transfusion bag: 1 mL placed in a cryovial and stored at −80°C for further DNA extraction and, a 3 mL aliquot used for plasma collection and cell counting (see flow cytometry paragraph below). The blood was transferred into a sterile 175 cm^2^ cell culture flask (Greiner Bio One, Germany), diluted up to 750 mL in PBS and distributed in sterile tubes with porous barriers (Leucosep™, Greiner Bio One Cat. 227 290). PBMCs and granulocytes were separated by density centrifugation on Ficoll-Paque Plus™ (GE Healthcare, Sweden) at 400 *g*, 20°C during 30 minutes. PBMCs were washed (3x) in PBS, re-suspended in 0.5% BSA-PBS, 2 mM EDTA (pH 7.2) and then counted and analyzed for viability using trypan dye exclusion (Countess® Automated Cell Counter, Invitrogen, USA). Granulocytes were recovered from the cell pellet of the density gradient and incubated (2x) with red blood cell lysis buffer (150 mM NH_4_Cl, 100 mM KHCO_3_, 1 mM EDTA) at 4°C during 12 and 4 minutes, respectively. Afterwards, granulocytes were washed (3x) in PBS, re-suspended in 0.5% BSA-PBS, 2 mM EDTA (pH 7.2), counted and evaluated for viability. An aliquot of PBMCs and granulocytes (5×10^6^ cells, each) was stored at −80°C for further DNA extraction. The remaining PBMCs were distributed according to the input requirements for magnetic-activated cell sorting (MACS, Miltenyi Biotech, Germany) to obtain T cells, B cells, monocytes, and NK cells. These populations were purified by positive selection using anti-CD4, anti-CD8, anti-CD19, anti-CD14, and anti-CD56 antibodies coupled to paramagnetic beads (Miltenyi Biotech, Germany). Granulocytes were split in two fractions for neutrophil and eosinophil purification. Neutrophils were obtained by positive selection using anti-CD16 antibodies and eosinophils were obtained using the untouched protocol (Eosinophil Isolation kit II, Miltenyi Biotech, Germany). Cell separations were done according to the manufacturers' instructions on LS columns. After purification, all populations were counted, aliquoted at ∼5×10^6^ cells per vial, snap frozen and stored at −80°C until DNA isolation. A replicate of 0.1–0.5×10^6^ cells was taken from each population to validate purities by flow cytometry.

### Flow cytometry

The purity of the populations was evaluated by immunophenotyping using four-color antibody panels (Table S4). Cells were re-suspended in FACS buffer (0.1% BSA in PBS) at a final concentration of 0.1×10^6^ cells per tube. Fc receptors were blocked with 1 µl of normal mouse serum (Cat. X0910, Dako Cytomanion, Denmark) during 10 minutes at 4°C. Optimized panels of fluorochrome-conjugated monoclonal antibodies were added to the cells, in a final volume of 100 µl and incubated for 30 minutes at 4°C. Every staining included the unstained sample and the corresponding panels of isotype controls to set the gates for positive and negative populations. After staining, cells were washed and fixed in 200 µl of 2% formaldehyde in PBS. Data were acquired using a FACS Calibur (BD Biosciences, USA), to at least 5000 events per population and analyzed by Flow Jo v7.6.3 (Tree Star Inc., USA). The achieved purities per cell population are presented in Tables S1 and S2.

### DNA extraction, bisulfite treatment and DNA methylation measurement

Genomic DNA was isolated from cell pellets using the QIAmp DNA Micro Kit (QIAGEN, Germany) according to the manufacturer's instructions. DNA concentration was measured by spectrophotometry (NanoDrop ND-1000 Spectrometer, Thermo Scientific, USA) and DNA quality was estimated with the ratio of absorbance A260/A280. Sixty samples of genomic DNA [500 ng] were bisulfite converted with the EZ-96 DNA Methylation Kit (Zymo Research Corporation, USA) according to the manufacturer's instructions. Array-based specific DNA methylation analysis was performed with the Infinium Human Methylation 450K bead chip technology (Illumina, USA). All samples were analyzed for more than 450,000 CpG sites at single nucleotide resolution with 99% coverage of RefSeq genes and 96% coverage of CpG islands. Probes were distributed in CpG island shelves, CpG island shores, CpG islands, promoter regions 5′UTRs, first exon, gene body and 3′UTRs. Additional CpG sites previously described as differentially methylated across tissue types, associated with tumors, located in FANTOM4 promoters, in DNase hypersensitive sites and/or miRNA promoter regions were also analyzed. Bisulfite-treated genomic DNA was whole-genome amplified, hybridized to HumanMethylation450 BeadChips (Illumina, USA) and scanned using the Illumina iScan at the Bioinformatic and Expression Analysis (BEA) Core Facility of the Karolinska Institutet. The intensity of the images was extracted with the GenomeStudio Methylation Software Module (v 1.9.0, Illumina, USA). The Infinium methylation data are available in the Gene Expression Omnibus (GEO) database (http://www.ncbi.nlm.nih.gov/geo/), under accession number GSE35069. Data and illustration tool for methylation at user-specified locations are also available on the website: http://publications.scilifelab.se/kere_j/methylation.

### QC analysis and data validation

Quality control was conducted in GenomeStudio software (v2011.1) using the methylation module (v1.9.0) according to the manufacturer's recommendations (Illumina, USA). Briefly, the controls included assessment of DNP and Biotin staining, hybridization, target removal, extension, bisulfite conversion, G/T mismatch, and negative and non-polymorphic controls. The various controls indicated overall good quality of DNA preparations and chip performances.

### Bioinformatics analysis

The raw data were imported into R v. 2.13 and analyzed with the Bioconductor lumi package [44]. The data was further visually inspected for quality check, adjusted for color channel imbalance and background noise, and normalized according to the quantile method. Probe-wise differential methylation was assayed by linear model and, subsequently, the pair-wise comparisons of each cell type versus whole blood were tested by Bayesian moderated t-test on the normalized matrix of intensity values (M values) [45]. The M-value is calculated as the log2 ratio of the intensities of methylated probe versus unmethylated probe and describes a measurement of how much more a probe is methylated compared to unmethylated [46]. A value close to 0 indicates a similar intensity between the methylated and unmethylated probes, which means the CpG site is about half-methylated [46]. Positive M-values mean that more molecules are methylated than unmethylated, while negative M-values mean that more molecules are unmethylated. The M values give higher resolution than the beta values for extreme methylation levels, whereas at low methylation levels they are colinear. Probes with Benjamini and Hochberg corrected p-value <0.01 were considered to be significant. Moreover, probe-wise methylation status (*i.e.* “Methylated”, “Marginal”, “Unmethylated”) was computed by fitting the normalized data to a two component Gamma mixture model. In brief, in each sample separately, the ratio between Cy3 and Cy5 signal for each probe defines the status (methylation %). The gamma fit model returns the probability that this signal ratio is balanced (“marginal”) or otherwise shifted towards Cy3 (“unmethylated”) or Cy5 (“methylated”) signal. In each pair-wise comparison, the significant probes with different methylation calls between each cell type and whole blood were considered for further analysis.

### Gene ontology analysis

Gene ontology enrichment of genes containing differentially methylated CpG sites was performed using The Database for Annotation, Visualization and Integrated Discovery v6.7 (DAVID, http://david.abcc.ncifcrf.gov) [47]. Analyses were based on a hypergeometric test with a p-value <0.01. Categories with a p-value <0.05 were considered significant and the top ten pathways are presented. Analyses were performed on the lists of genes showing unmethylated state in a selected cell population and methylated in whole blood using the whole human genome as background.

## Supporting Information

Figure S1 Plots showing the purity of sorted cells from whole blood by flow cytometry.
(DOCX)

Figure S2 Distributions of the mean M-values and standard deviations for the analyzed probes for the six individuals for all ten cell populations.
(DOCX)

Figure S3 Venndiagrams for differentially methylated CpG sites compared between peripheral blood mononuclear cells (PBMC) and granulocytes. Data is based on a linear model comparing the two principal cell populations to whole blood using M-values. The data was then subjected to a gamma fit model in order to group the data into the defined calls: unmethylated, margin and methylated.
(DOCX)

Table S1 Composition (%) of whole blood, peripheral blood mononuclear cells (PBMC) and granulocyte fractions as determined by flow cytometry.
(DOCX)

Table S2 Composition (%) of sorted cell populations from whole blood including T cells, NK cells, B cells, monocytes, neutrophils and eosinophils, as determined by flow cytometry.
(DOCX)

Table S3 Differentially methylated probes (n = 1865) in isolated cells from blood in selected susceptibility genes for inflammatory diseases from the catalog of published genome-wide association studies (http://www.genome.gov/gwastudies/) [30]. The included diseases were asthma, atopy, atopic dermatitis, inflammatory bowel disease, rheumatoid arthritis, systemic lupus erythematosus, Type 1 and Type 2 diabetes.
(XLSX)

Table S4 Antibodies used for flow cytometry panels.
(DOCX)
