# Supplementary figures and images for: Differential DNA Methylation in Purified Human Blood Cells: Implications for Cell Lineage and Studies on Disease Susceptibility
Source: PLoS One. 2012 Jul 25;7(7):e41361. doi: 10.1371/journal.pone.0041361 (PMC3405143; doi:10.1371/journal.pone.0041361)

**Figure S1**


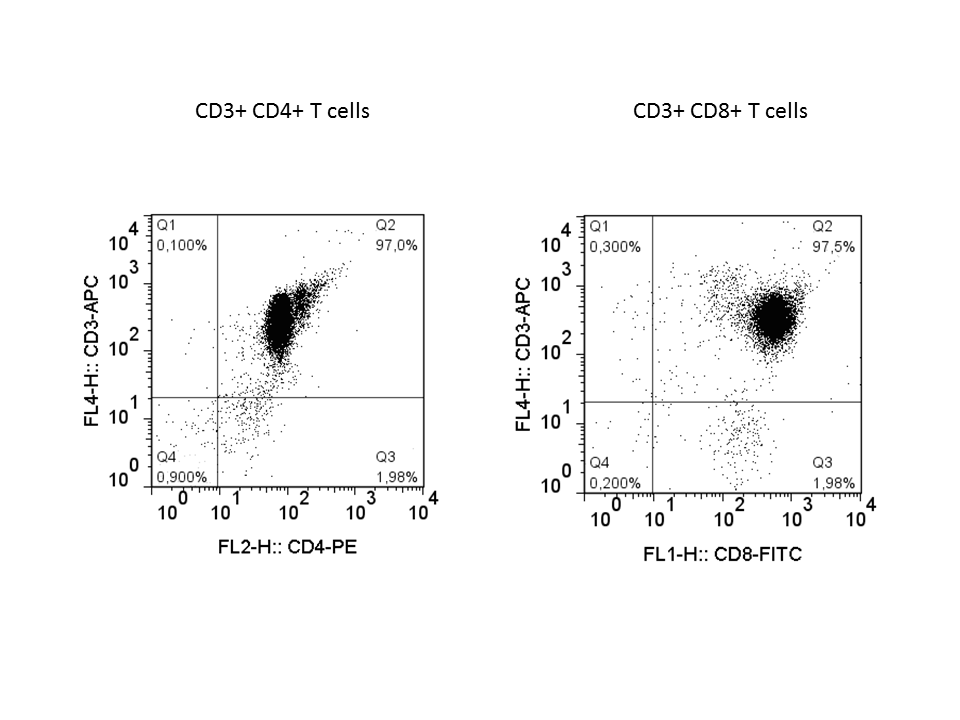


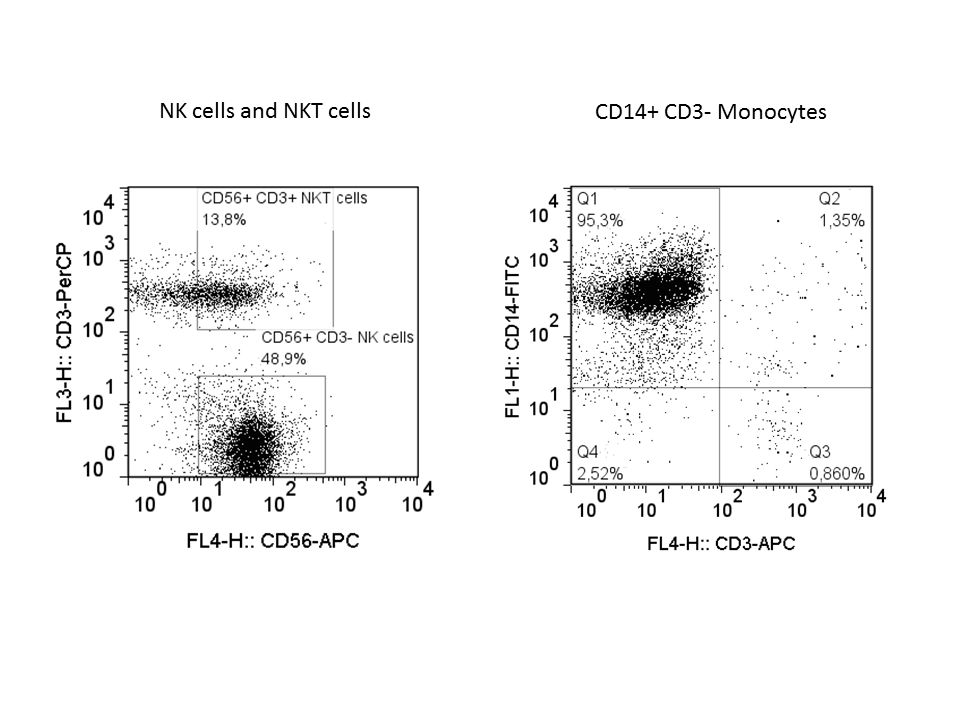


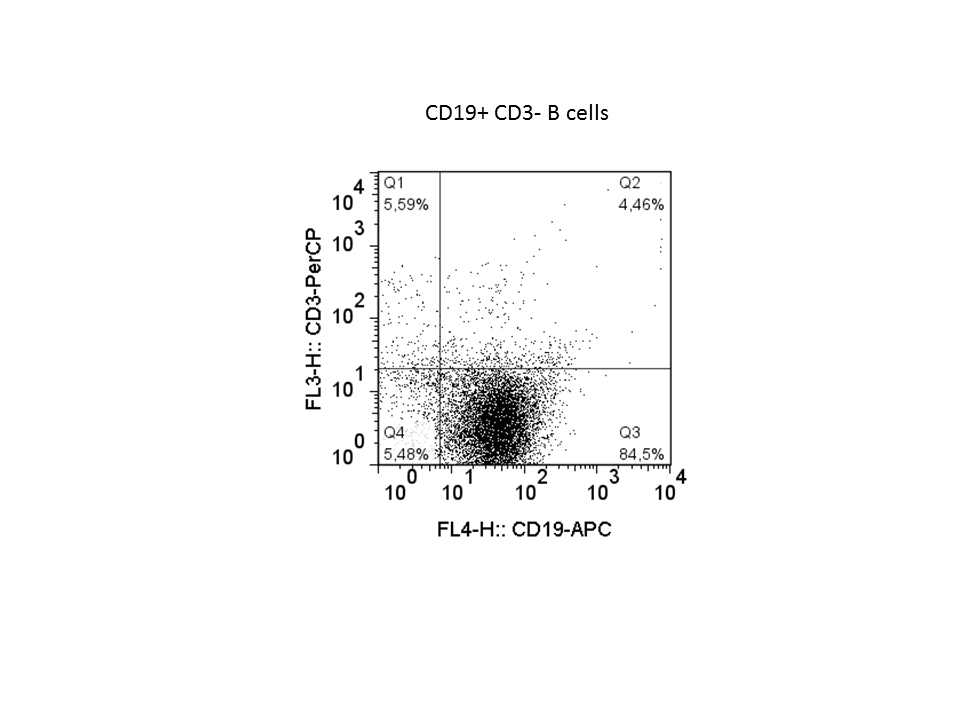

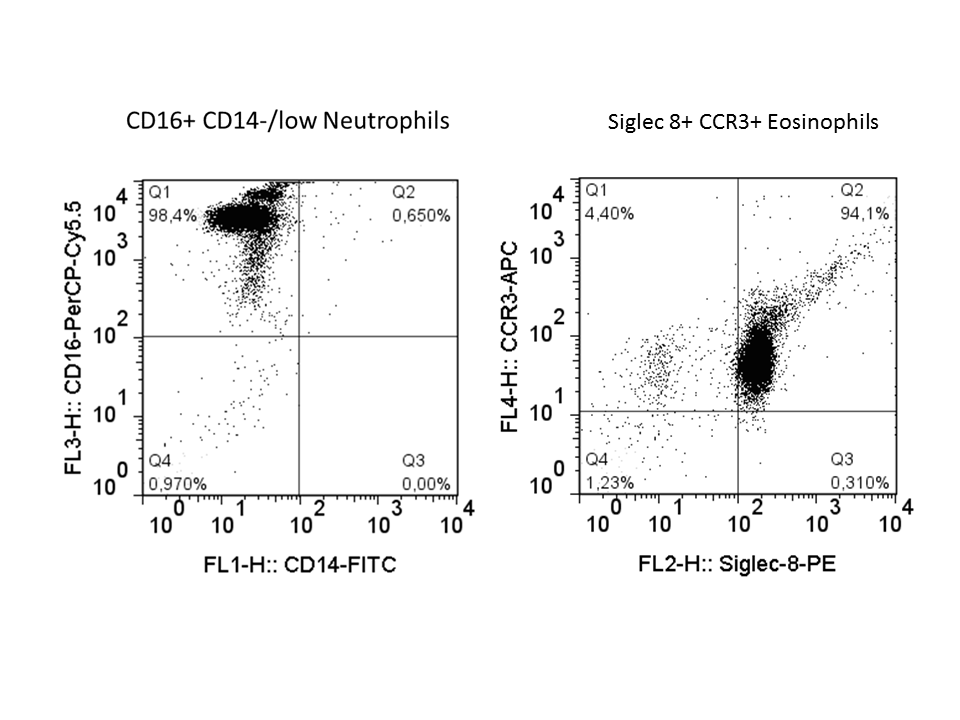

Supplement: Figure S1 — Plots showing the purity of sorted cells from whole blood by flow cytometry. (DOCX) [file pone.0041361.s001.docx]

**Figure S2**

**
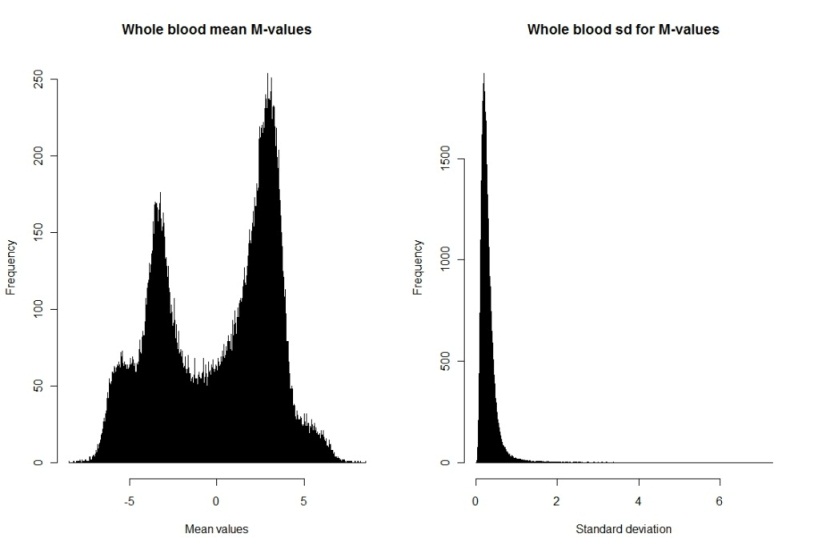

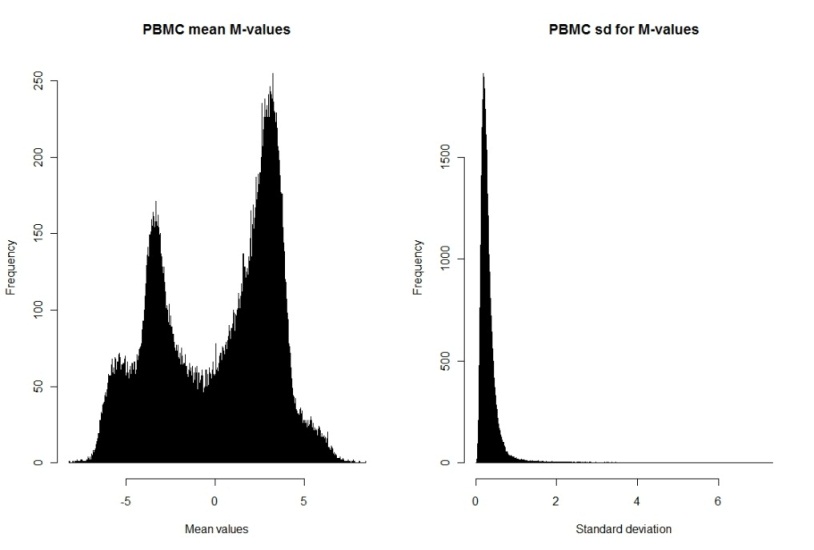

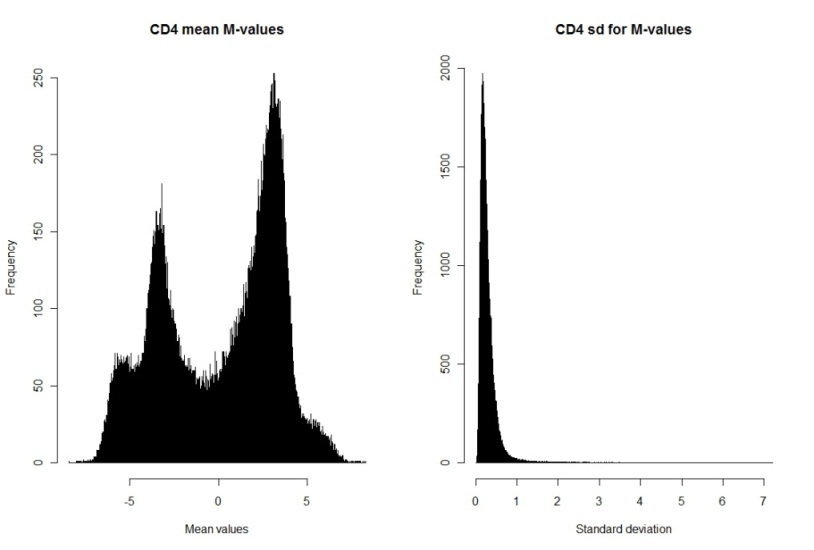

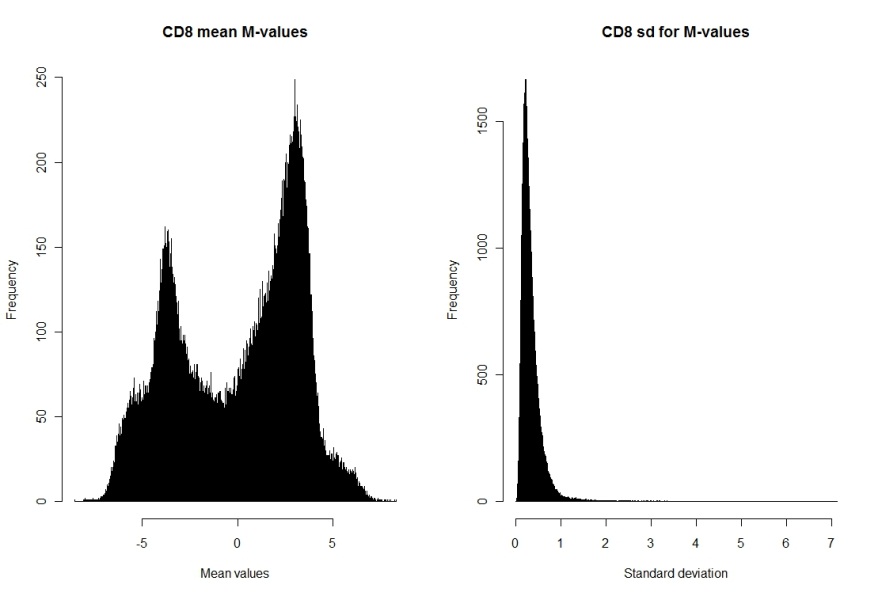

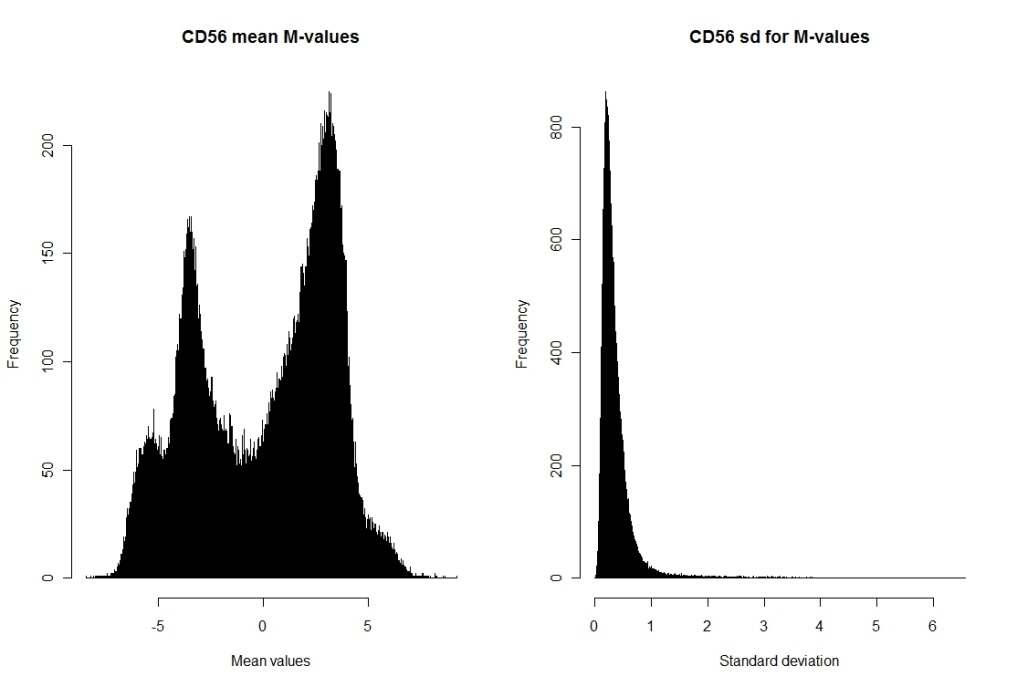

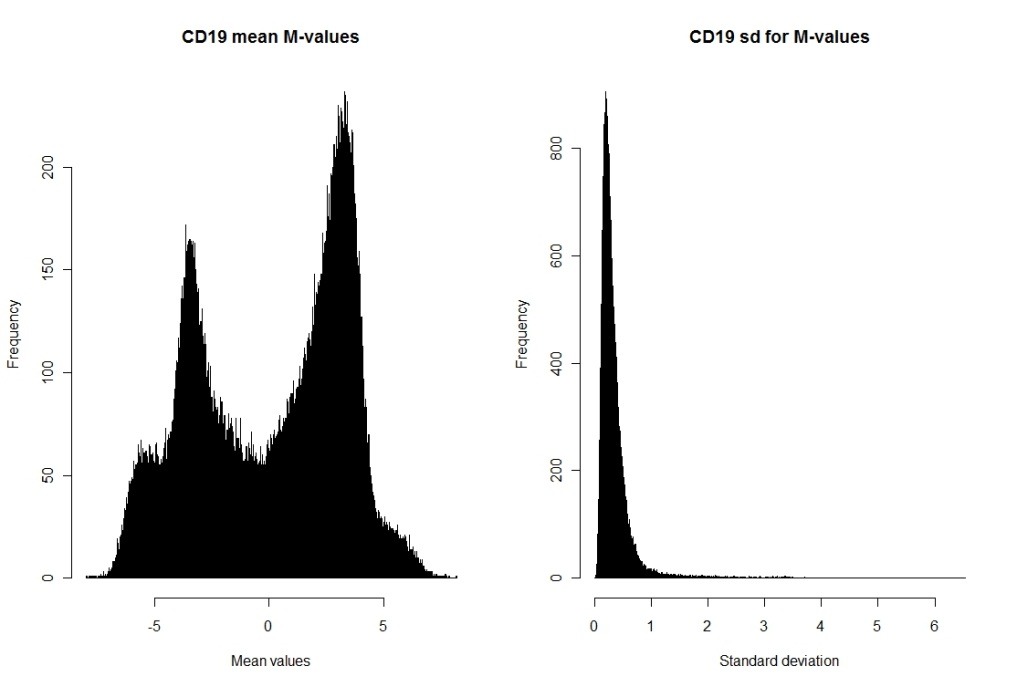

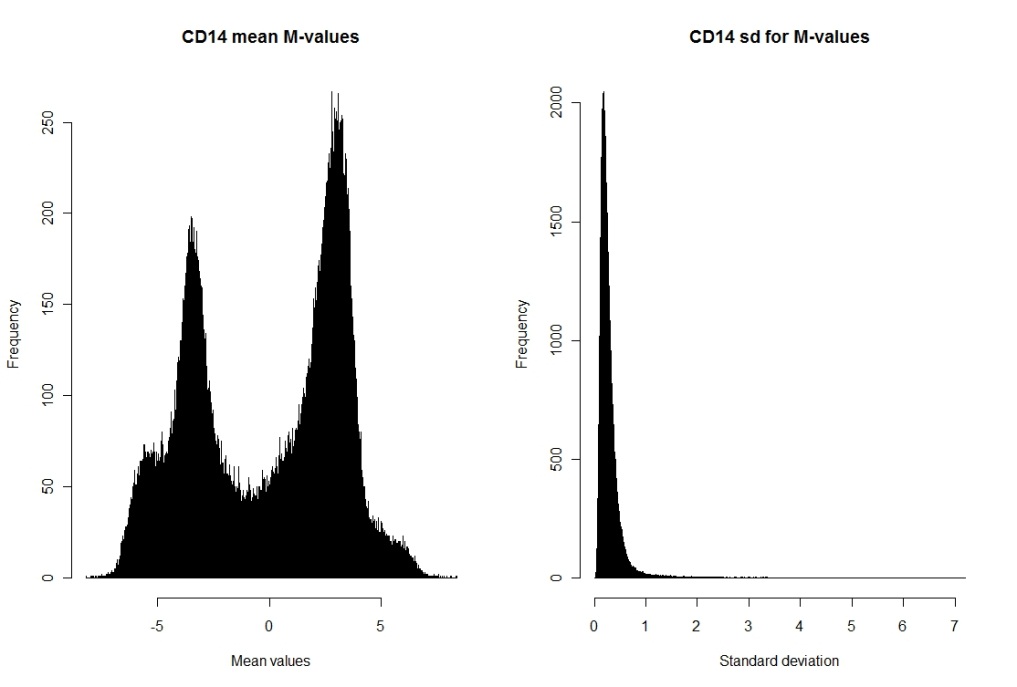

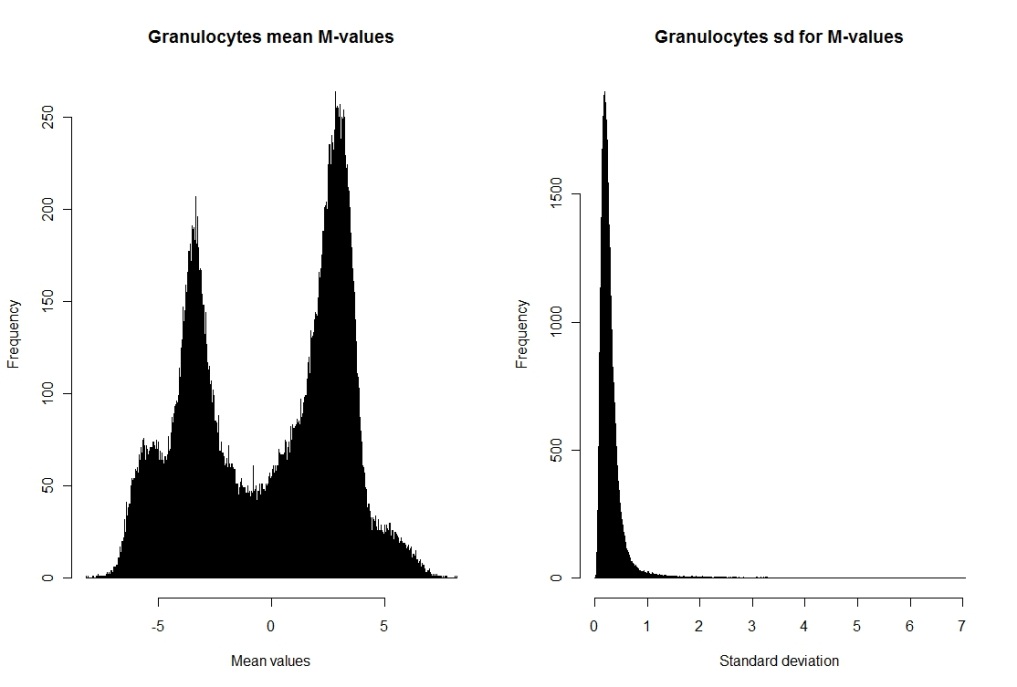

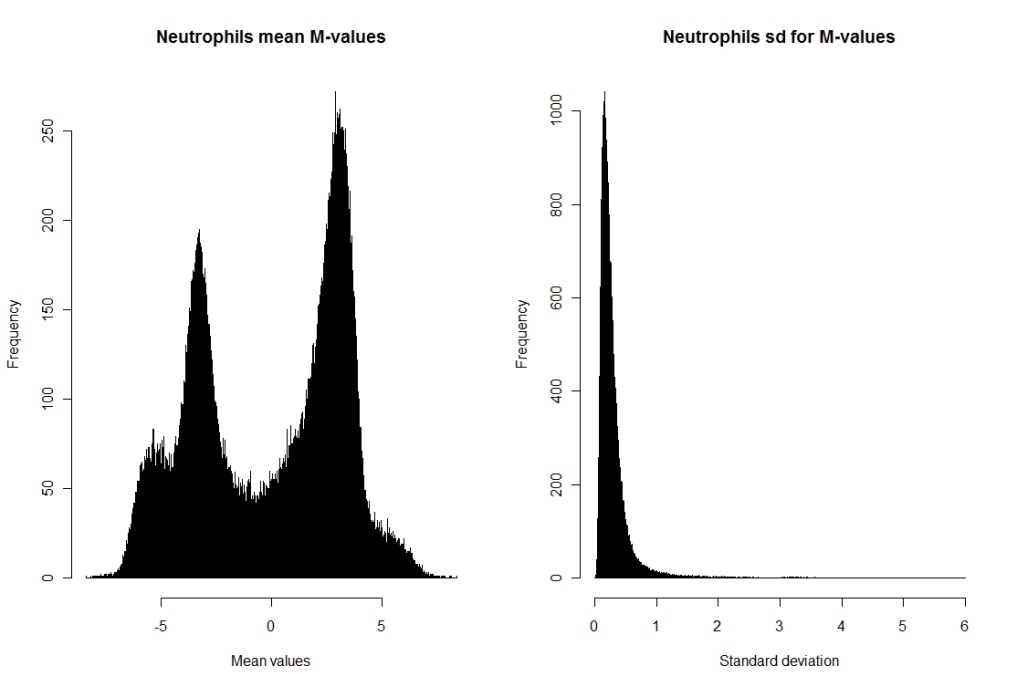

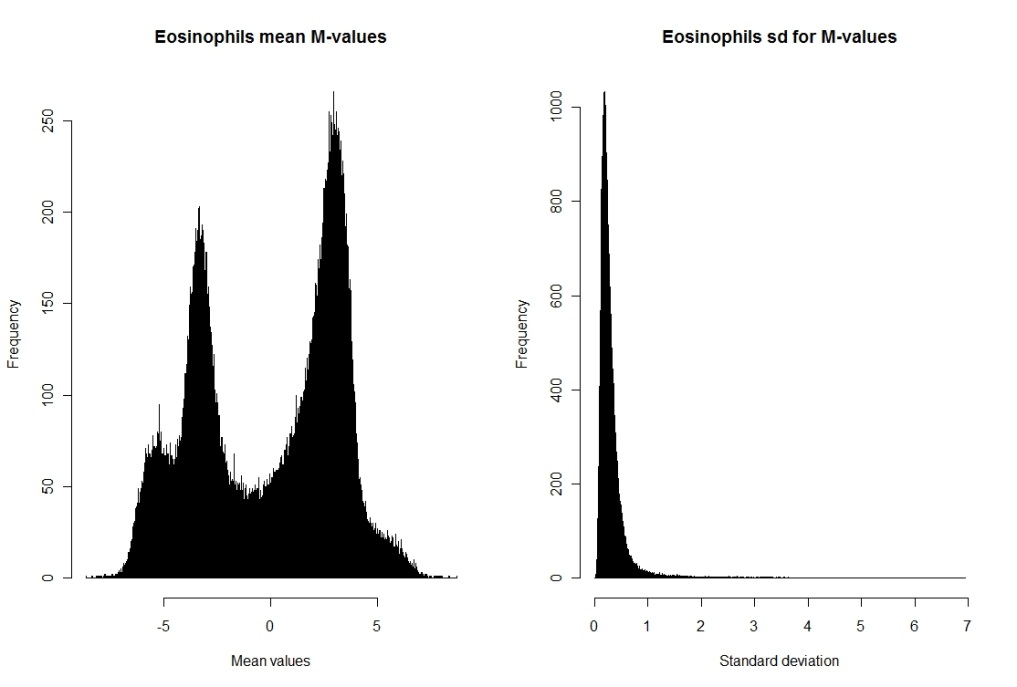
**

Supplement: Figure S2 — Distributions of the mean M-values and standard deviations for the analyzed probes for the six individuals for all ten cell populations. (DOCX) [file pone.0041361.s002.docx]
