# Supplementary material for: Differential DNA Methylation in Purified Human Blood Cells: Implications for Cell Lineage and Studies on Disease Susceptibility
Source: PLoS One. 2012 Jul 25;7(7):e41361. doi: 10.1371/journal.pone.0041361 (PMC3405143; doi:10.1371/journal.pone.0041361)
Supplement: Figure S3 — Venndiagrams for differentially methylated CpG sites compared between peripheral blood mononuclear cells (PBMC) and granulocytes. Data is based on a linear model comparing the two principal cell populations to whole blood using M-values. The data was then subjected to a gamma fit model in order to group the data into the defined calls: unmethylated, margin and methylated. (DOCX) [file pone.0041361.s003.docx]

**Figure S3**

**Methylated probes**

**Unmethylated probes**

**All probes**

11201

3847

1975

747

13023

622

9038

13177

14188

**PBMC**

**Granulocytes**
