# Supplementary material for: Differential DNA Methylation in Purified Human Blood Cells: Implications for Cell Lineage and Studies on Disease Susceptibility
Source: PLoS One. 2012 Jul 25;7(7):e41361. doi: 10.1371/journal.pone.0041361 (PMC3405143; doi:10.1371/journal.pone.0041361)
Supplement: Table S1 — Composition (%) of whole blood, peripheral blood mononuclear cells (PBMC) and granulocyte fractions as determined by flow cytometry. (DOCX) [file pone.0041361.s004.docx]

**Table S1.** Composition (%) of whole blood, peripheral blood mononuclear cells (PBMC) and granulocyte fractions as determined by flow cytometry.

| Donor # | CD4^+^CD3^+^ | CD8^+^CD3^+^ | CD14^+^ | CD19^+^ | CD56^+^CD3^-^ | CD16^+^ | Siglec-8^+^ |
| --- | --- | --- | --- | --- | --- | --- | --- |
| *Whole Blood* |  |  |  |  |  |  |  |
| 1 | 13.4 | 11.6 | 5.8 | 2.1 | 4.0 | 55.0 | 3.3 |
| 2 | 14.0 | 3.9 | 10.1 | 3.0 | 3.4 | 62.0 | 4.9 |
| 3 | 11.2 | 4.1 | 1.3 | 1.9 | 1.0 | 73.7 | 3.7 |
| 4 | 11.3 | 5.6 | 2.6 | 4.3 | 1.6 | 68.6 | 5.1 |
| 5 | 11.3 | 3.6 | 5.3 | 1.6 | 0.7 | 75.9 | 1.1 |
| 6 | 19.3 | 8.0 | 7.3 | 5.2 | 3.9 | 54.7 | 4.7 |
| Mean ± SD | 13.4 ± 3.12 | 6.13 ± 3.13 | 5.40 ± 3.17 | 3.01 ± 1.44 | 2.43 ± 1.50 | 64.9 ± 9.19 | 3.8 ± 1.5 |
|  |  |  |  |  |  |  |  |
| *PBMC* |  |  |  |  |  |  |  |
| 1 | 27.6 | 19.4 | 7.2 | 4.1 | 9.7 | 26.2 | 0.8 |
| 2 | 35.7 | 7.6 | 22.4 | 10.5 | 9.9 | 18.6 | 0.4 |
| 3 | 51.9 | 12.9 | 10.2 | 5.0 | 5.4 | 7.3 | 1.3 |
| 4 | 39.8 | 18.3 | 6.9 | 8.3 | 6.8 | 19.8 | 0.2 |
| 5 | 47.9 | 8.6 | 5.3 | 8.5 | 3.5 | 21.7 | 0.6 |
| 6 | 38.8 | 13.5 | 9.6 | 10.9 | 9.4 | 24.8 | 0.6 |
| Mean ± SD | 40.2 ± 8.68 | 13.3 ± 4.83 | 10.2 ± 6.21 | 7.88 ± 2.79 | 7.45 ± 2.64 | 19.7 ± 6.74 | 0.65 ± 0.37 |
|  |  |  |  |  |  |  |  |
| *Granulocyte* |  |  |  |  |  |  |  |
| 1 | 3.4 | 2.9 | 0.4 | 0.1 | 0.5 | 88.0 | 5.3 |
| 2 | 4.6 | 2.2 | 0.7 | 0.3 | 0.5 | 85.4 | 5.8 |
| 3 | 2.6 | 1.2 | 0.1 | 0.1 | 0.0 | 89.3 | 5.1 |
| 4 | 1.5 | 0.6 | 0.3 | 0.3 | 0.1 | 90.7 | 5.7 |
| 5 | 1.6 | 0.6 | 0.4 | 0.2 | 0.1 | 95.6 | 1.2 |
| 6 | 3.0 | 1.4 | 0.3 | 0.2 | 0.2 | 91.1 | 3.4 |
| Mean ± SD | 2.78 ± 1.16 | 1.48 ± 0.91 | 0.36 ± 0.19 | 0.20 ± 0.08 | 0.23 ± 0.21 | 90 ± 3.42 | 4.41 ± 1.79 |

SD - standard deviation, PBMC - peripheral blood mononuclear cells
