# Supplementary material for: Differential DNA Methylation in Purified Human Blood Cells: Implications for Cell Lineage and Studies on Disease Susceptibility
Source: PLoS One. 2012 Jul 25;7(7):e41361. doi: 10.1371/journal.pone.0041361 (PMC3405143; doi:10.1371/journal.pone.0041361)
Supplement: Table S2 — Composition (%) of sorted cell populations from whole blood including T cells, NK cells, B cells, monocytes, neutrophils and eosinophils, as determined by flow cytometry. (DOCX) [file pone.0041361.s005.docx]

**Table S2.** Composition (%) of sorted cell populations from whole blood including T cells, NK cells, B cells, monocytes, neutrophils and eosinophils, as determined by flow cytometry.

|  | CD4^+^ T cells | | CD8^+^ T cells | | CD14^+^ monocytes | | CD19^+^ B cells | | CD56^+^ NK cells | | Neutrophils | | Eosinophils |
| --- | --- | --- | --- | --- | --- | --- | --- | --- | --- | --- | --- | --- | --- |
| Donor # | CD4^+^ | CD4^+^  CD3^+^ | CD8^+^ | CD8^+^  CD3^+^ | CD14^+^ | CD14^+^  CD3^-^ | CD19^+^ | CD19^+^  CD3^-^ | CD56^+^ | CD56^+^  CD3^-^ | CD16^+^ | CD16^+^  CD3^-^ | Siglec-8^+^  CCR3^+^ |
| 1 | 97.4 | 93.6 | 91.1 | 93.2 | 96.6 | 95.4 | 87.7 | 89.5 | 73.5 | 57.5 | 98.9 | 97.7 | 89.4 |
| 2 | 96.0 | 94.2 | 85.4 | 88.6 | 95.5 | 95.1 | 94.1 | 92.8 | 83.5 | 73.0 | 98.5 | 98.4 | 83.6 |
| 3 | 95.0 | 93.5 | 96.0 | 97.6 | 79.9 | 77.7 | 72.2 | 67.9 | 75.0 | 42.2 | 94.8 | 94.6 | 45.7 |
| 4 | 98.4 | 97.5 | 99.1 | 99.2 | 93.2 | 91.4 | 91.0 | 86.2 | 77.0 | 59.8 | 98.6 | 98.5 | 96.4 |
| 5 | 98.9 | 98.1 | 99.1 | 99.4 | 98.6 | 97.2 | 93.0 | 85.5 | 90.5 | 60.2 | 99.3 | 98.9 | 80.0 |
| 6 | 98.5 | 96.7 | 98.9 | 99.1 | 96.8 | 95.3 | 82.6 | 73.9 | 94.0 | 75.7 | 97.1 | 96.6 | 96.1 |
|  |  |  |  |  |  |  |  |  |  |  |  |  |  |
| Mean ±  SD | 97.3 ± 1.5 | 95.6 ± 2.0 | 94.9±5.6 | 96.1 ± 4.3 | 93.4 ± 6.8 | 92.0 ± 7.26 | 86.7 ± 8.2 | 82.6 ± 9.6 | 82.2 ± 8.2 | 61.4 ± 12 | 97.8 ± 1.67 | 97.4 ± 1.61 | 81.8 ± 18.8 |

SD - Standard deviation
