# Supplementary material for: Differential DNA Methylation in Purified Human Blood Cells: Implications for Cell Lineage and Studies on Disease Susceptibility
Source: PLoS One. 2012 Jul 25;7(7):e41361. doi: 10.1371/journal.pone.0041361 (PMC3405143; doi:10.1371/journal.pone.0041361)
Supplement: Table S4 — Antibodies used for flow cytometry panels. (DOCX) [file pone.0041361.s007.docx]

**Supplementary Table S4.** Antibodies used for flow cytometry panels.

| **Antibody panels for whole blood, PBMCs and granulocyte fractions*** | | | | | | | | | |
| --- | --- | --- | --- | --- | --- | --- | --- | --- | --- |
|  | **Panel 1** | | | **Panel 2** | | | | **Isotype mix** | |
| FL1 FITC | CD14 (HCD14, eBioscience 325604) | | | CD8 (RPA-T8, IMG-5917C) | | | | IgG_1_ κ (MOPC-21, eBioscience 400110) | |
| FL2 PE | Siglec-8 (7C9, eBioscience 347104 ) | | | CD4 (RPA-T4, IMG-5916D) | | | | IgG_1_ κ (MOPC-21, eBioscience 400114) | |
| FL3 PerCP-Cy5.5 | CD16 (3G8, eBioscience 302028) | | | CD3 (UCHT1, eBioscience 300430) | | | | IgG_1_ κ (MOPC-21, eBioscience 400150) | |
| FL4 APC | CD19 (HIB19, eBioscience 302212) | | | CD56 (B159, BD 555518) | | | | IgG_1_ κ (MOPC-21, eBioscience 400122) | |
|  | | | | | | | | | |
| **Antibody panels for purified populations** | | | | | | | | | |
|  | **CD4** | **CD8** | **CD56** | | **CD19** | **CD14** | **CD16** | | **Eosinophils** |
| FL1 FITC | CD14 | CD8 |  | |  | CD14 | CD14 | |  |
| FL2 PE | CD4 |  |  | |  |  |  | | Siglec-8 |
| FL3 PerCP-Cy5.5 |  |  | CD3 | | CD3 | CD16 | CD16 | |  |
| FL4 APC | CD3  (UCHT1, eBioscience 300412) | CD3 | CD56 | | CD19 | CD3 | CD3 | | CCR3  (MG2b-57, eBioscience 310708) |
|  | | | | | | | | | |
| **Mixes for isotype controls** | | | | | | | | | |
| FL1 FITC | IgG_1_ | IgG_1_ |  | |  | IgG_1_ | | IgG_1_ |  |
| FL2 PE | IgG_1_ |  |  | |  |  | |  | IgG_1_ |
| FL3 PerCP-Cy5.5 |  |  | IgG_1_ | | IgG_1_ | IgG_1_ | | IgG_1_ |  |
| FL4 APC | IgG_1_ | IgG_1_ | IgG_1_ | | IgG_1_ | IgG_1_ | | IgG_1_ | IgG_2_b  (MG2b-57, eBioscience 401210) |

*Target surface antigens (clone and catalog number)

The following two staining panels were used for whole blood, PBMCs and granulocytes:

Anti-CD14 FITC, anti-Siglec-8 PE, anti-CD16 PerCP-Cy5.5, and anti-CD19 APC (panel 1); and, anti-CD8 FITC, anti-CD4 PE, anti-CD3 PerCP-Cy5.5, and anti-CD56 APC (panel 2).

For every sorted population a specific staining panel was used to determine the purity:

Anti-CD14 FITC, anti-CD4 PE and anti-CD3 APC (CD4 positive selection)

Anti-CD8 FITC and anti-CD3 APC (CD8 positive selection)

Anti-CD14 FITC, anti-CD16 PerCP-Cy5.5 and anti-CD3 APC (CD14 positive selection)

Anti-CD3 PerCP-Cy5.5 and anti-CD19 APC (CD19 positive selection)

Anti-CD3 PerCP-Cy5.5 and anti-CD56 APC (CD56 positive selection)

Anti-CD14 FITC, anti-CD16 PerCP-Cy5.5 and anti-CD3 APC (CD16 positive selection)

Anti-Siglec-8 PE and anti-CCR3 APC (eosinophils untouched).
